# Hyperactivity and Hypermotivation Associated With Increased Striatal mGluR1 Signaling in a Shank2 Rat Model of Autism

**DOI:** 10.3389/fnmol.2018.00107

**Published:** 2018-06-19

**Authors:** Meera E. Modi, Julie M. Brooks, Edward R. Guilmette, Mercedes Beyna, Radka Graf, Dominik Reim, Michael J. Schmeisser, Tobias M. Boeckers, Patricio O’Donnell, Derek L. Buhl

**Affiliations:** ^1^Pfizer Internal Medicine Research Unit, Pfizer Inc., Cambridge, MA, United States; ^2^Institute for Anatomy and Cell Biology, Ulm University, Ulm, Germany; ^3^Division of Neuroanatomy, Institute of Anatomy, Otto-von-Guericke University, Magdeburg, Germany; ^4^Leibniz Institute for Neurobiology, Magdeburg, Germany

**Keywords:** SHANK, Shank2, autism spectrum disorders, rat model, mGluR, motivation

## Abstract

Mutations in the *SHANK* family of genes have been consistently identified in genetic and genomic screens of autism spectrum disorder (ASD). The functional overlap of *SHANK* with several other ASD-associated genes suggests synaptic dysfunction as a convergent mechanism of pathophysiology in ASD. Although many ASD-related mutations result in alterations to synaptic function, the nature of those dysfunctions and the consequential behavioral manifestations are highly variable when expressed in genetic mouse models. To investigate the phylogenetic conservation of phenotypes resultant of *Shank2* loss-of-function in a translationally relevant animal model, we generated and characterized a novel transgenic rat with a targeted mutation of the *Shank2* gene, enabling an evaluation of gene-associated phenotypes, the elucidation of complex behavioral phenotypes, and the characterization of potential translational biomarkers. The *Shank2* loss-of-function mutation resulted in a notable phenotype of hyperactivity encompassing hypermotivation, increased locomotion, and repetitive behaviors. Mutant rats also expressed deficits in social behavior throughout development and in the acquisition of operant tasks. The hyperactive phenotype was associated with an upregulation of mGluR1 expression, increased dendritic branching, and enhanced long-term depression (LTD) in the striatum but opposing morphological and cellular alterations in the hippocampus (HP). Administration of the mGluR1 antagonist JNJ16259685 selectively normalized the expression of striatally mediated repetitive behaviors and physiology but had no effect on social deficits. Finally, *Shank2* mutant animals also exhibited alterations in electroencephalography (EEG) spectral power and event-related potentials, which may serve as translatable EEG biomarkers of synaptopathic alterations. Our results show a novel hypermotivation phenotype that is unique to the rat model of *Shank2* dysfunction, in addition to the traditional hyperactive and repetitive behaviors observed in mouse models. The hypermotivated and hyperactive phenotype is associated with striatal dysfunction, which should be explored further as a targetable mechanism for impairment in ASD.

## Introduction

Mutations within the *SHANK* family of genes (comprising *SHANK1*, *PROSAP1/SHANK2* and *PROSAP2/SHANK3*) are over-represented in the autism spectrum disorder (ASD) population and are the cause of specific disorders such as Phelan-McDermid Syndrome (Jiang and Ehlers, 2013). Inherited and *de novo* *SHANK2* mutations have been identified in several independent families with ASD (Berkel et al., 2010; Leblond et al., 2012; Monteiro and Feng, 2017), many of which lie in functional binding domains for proteins interacting with SHANK2 within the synapse, making *SHANK2* mutation a representative model of the synaptopathy observed in ASD (Berkel et al., 2012; Jiang and Ehlers, 2013).

Synaptopathic consequences of *Shank* mutations are particularly evident in striatal function. Dysregulation of striatal circuits resulting in abnormal social motivation has been hypothesized as a mechanism for the behavioral characteristics in ASD (Chevallier et al., 2012, 2016). Accumulating evidence has also implicated the striatum in the phenomenology of repetitive behaviors (Kohls et al., 2014). Further, the corticolimbic-ventro-striatal network is dysregulated in patients with ASD and obsessive-compulsive disorder (Ameis and Catani, 2015; Wood and Ahmari, 2015; Bariselli et al., 2016). Given its role in both social motivation and production of motivated and habitual repetitive behaviors, the striatum may consequently serve as a locus for the pathogenic processes that result in the symptomatology of ASD.

Mutation of *Shank3* results in altered dendritic arborization, synaptic transmission, postsynaptic density (PSD) composition and excitatory/inhibitory balance in medium spiny neurons (MSNs) of the striatum (Peca et al., 2011; Filice et al., 2016; Jaramillo et al., 2016; Mei et al., 2016; Wang et al., 2016; Reim et al., 2017; Vicidomini et al., 2017), as well as hypoactivity (Kouser et al., 2013; Speed et al., 2015) and perseverative overgrooming (Kouser et al., 2013; Speed et al., 2015; Jaramillo et al., 2016, 2017), linking repetitive behaviors to altered striatal physiology (Dhamne et al., 2017; Kabitzke et al., 2018). *Shank3* mutation impairs the striatopallidal synapses of striatal MSNs and enhancement of their activity decreases overgrooming (Wang et al., 2017). Mutation of *Shank2* has been similarly implicated in repetitive behaviors in mice; however, *Shank2* mutation primarily manifests as hyperactivity (Schmeisser et al., 2012; Won et al., 2012) as opposed to the hypoactivity observed in multiple *Shank3* mouse models (Kouser et al., 2013; Speed et al., 2015; Jaramillo et al., 2016). *Shank2* mutant mice have substantially increased locomotor activity relative to wild type mice and engage in a repetitive jumping behavior at the expense of species-typical digging behaviors (Schmeisser et al., 2012; Won et al., 2012). Accompanying the hyperactive behavior is an upregulation of ionotropic glutamate receptors, most broadly within the striatum in the *Shank2* mutant mouse (Schmeisser et al., 2012). Together, these findings indicate that in the mouse, mutation of *Shank* genes results in functional changes in the striatum that are likely associated with prominent repetitive and hyperactive phenotypes.

The neural circuits underlying motivation and repetitive behaviors have been historically characterized in rat models due to their ability to rapidly acquire complex, striatally mediated tasks (Hart et al., 2014; Jaramillo and Zador, 2014). To explore the relationship between the striatal cellular and molecular consequences of *Shank2* mutation and the repetitive and motivational phenotypes associated with striatal dysregulation in rats, we generated a novel transgenic *Shank2* mutant rat. This model has enabled the assessment of complex social and motivated behaviors and the identification of a potentially translational electroencephalography (EEG) based biomarker of neural circuit dysfunction that has not been shown in previous mouse models.

Similar to *Shank2* mutant mice, our rat model exhibits social and repetitive impairments and corresponding cellular alterations. *Shank2* mutant rats additionally have a profound phenotype of hyperactivity and hypermotivation that can be ameliorated through the administration of dopamine receptor 1 (D1R) or metabotropic glutamate receptor 1 (mGluR1) antagonists that normalize the observed electrophysiological alterations in the striatum. The characterization of phenotypes that are both consistent across species and amenable to pharmacological manipulation opens the door for the identification of drug treatment strategies for the impairments of ASD with high translational validity.

## Materials and Methods

### Model Generation

#### Animals

In collaboration with Horizon Discovery, we generated a transgenic Sprague-Dawley rat line expressing a targeted deletion of the *Shank2* gene that causes a frameshift and a premature stop (hereafter homozygous mutants referred to as KO; heterozygous, HET; and wild type, WT) using zinc finger nuclease technology. The deletion was designed to mimic one identified in ASD patients that disrupts the PDZ domain of the protein (Berkel et al., 2012). This study was carried out in accordance with the recommendations of the Guide for the Care and Use of Laboratory Animals. The protocol was approved by the Pfizer Institutional Animal Care and Use Committee.

#### Generation of Rats Carrying the Shank2 Deletion

**Figure 1:**
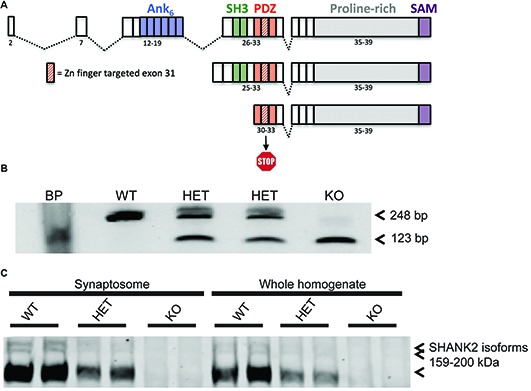
Generation of the *Shank2* mutant rat. **(A)** 437 bp genomic DNA (gDNA) deletion, encompassing exon 31 in the PDZ domain of the rat *Shank2* gene, results in a frameshift mutation and premature stop codon in the resultant mRNA transcript. **(B)** RT-PCR analysis of WT, HET and KO rat Shank2 mRNA around exon 31. Arrows indicate the WT (upper–248 bp) and KO (lower–123 bp) expected PCR products in the resultant Zn-finger KO mRNA. **(C)** No detectable expression of any of the three variants of full length SHANK2 protein product is detected in the KO animals in either whole brain homogenate or enriched synaptosomal fractions (see entire western blot in Supplementary Figure S1B).

A deletion was introduced using zinc finger nuclease technology. The endonucleases targeted the GACCGGG GACTTCTTGA TTGAGGTAGG ACACAGGTG sequence flanking the region of interest (Supplementary Figure S1A). Deletion was confirmed at both the genomic as well as mRNA level (Figure 1B; Supplementary Figure S1A). Two founder lines (Lines 8a and 13) were generated and initially characterized for consistency of phenotype in locomotor activity. Line 13 was maintained and used for all further experiments.

After the model was established, all animals were bred, genotyped, and raised until weaning (P21) at Charles River Laboratories (Wilmington, MA, USA). Experimental animals were produced by F1 HET × HET paired matings to produce litters containing all genotypes in Mendelian ratios. The F1 parents were generated from crosses between KO males and outbred Sprague-Dawley females. Prior to use, the genotype of all experimental animals was determined by Charles River genotyping service. Animals were shipped to Pfizer (Cambridge, MA, USA) between 21 days and 42 days. Because of intra-animal aggression observed in KO animals, all animals were singly housed after shipment in irradiated Innovive Caging (17’L × 13.4”W × 9.9”H) with Alpha-dry bedding with Bed-r’Nest and Nylabone enrichment. Animals were fed Lab Diet 5053 PicoLab Rodent Diet 20 and water *ad libitum*, except where otherwise noted. Animals were maintained at a temperature of 20–26°C, 30%–70% humidity with a 12:12 light:dark cycle.

#### Genotyping

Tail snips from rats were used for genomic DNA (gDNA) isolation. Tissue was digested using proteinase K and gDNA was isolated using Qiagen’s Blood and Tissue DNeasy kit. A total of 2 μl of gDNA was used for genomic PCR using the following primers: forward: 5′-TGGGTCAACACTGCTCTCTG-3′ and reverse: 5′-AGACTCCTCAAATGATCAAGCATTAC-3′. The expected PCR product sizes for WT and KO (exon 31 deleted) in Line 13 was 1171 and 501, respectively.

#### RT-PCR

Brain cortical RNA was isolated using Qiazol RNA isolation. Briefly, animals were euthanized humanely under approved animal use and care protocols. After dissection, total brains were flash frozen in liquid nitrogen and stored at −80°C until RNA isolation. Frozen brains were homogenized in Qiazol with a 5 mm bead in Tissuelyzer (Qiagen) for 5 min at 25 Hz and RNA isolation proceeding as per Qiagen protocol. Quantitated RNA was converted to cDNA using VILO Superscript cDNA synthesis reagent (Invitrogen). PCR amplification of WT or exon 31 deleted mRNA was performed with PCR primers E30: 5′-GAGGGCTTTGGATTTGTG-3′; and E32: 5′-GACGACCTTAAGGACGAG-3′ with the expected PCR products for WT and KO mRNA of 248 bp and 123 bp, respectively.

#### Western Blot

SHANK2 levels were analyzed from WT and KO animals from Line 13. One cortical hemisphere/animal from four male rats per genotype (at 7 weeks of age) were processed into filtered synaptosome fractions for analysis. In each lane, 30 μg of total protein was loaded on a 6% NuPAGE gel. The deletion of SHANK2 was confirmed using the SHANK2 (S23b-6) antibody from Novus Biologicals (#NBP1-44509), generated against the 84–309 amino acids of rat SHANK2 (SH3/PDZ domains) and does not cross-react with either SHANK1 or SHANK3, at a concentration of 1:4000 and normalized to the transferrin receptor (85 kDa) from (Invitrogen #13-6800) at a concentration of 1:2000.

### Behavioral Assays

Behavioral assays were performed in several cohorts of adult rats (>8 weeks of age, singly housed, male-unless otherwise noted) bred at Charles River Laboratories and tested at Pfizer. Cohort 1 was used for social investigation assays, including juvenile play (*N* = 8/genotype at 5–6 weeks), social recognition (*n* = 10/genotype at 8 weeks), and social approach (*N* = 13/genotype at 9 weeks). Cohort 2 was used for the open field assay to derive locomotion, rearing, and repetitive circling (*N* = 11/genotype). Cohort 3 was used for the operant conditioning assay (*N* = 8/genotype). Cohort 4 was used for the location discrimination touchscreen task (*N* = 5–8/genotype).

#### Juvenile Play

Juvenile male animals (5–6 weeks of age) of each genotype (KO, WT) were tested for the exhibition of play behaviors with WT age-matched male conspecifics. Experimental animals were marked and placed in a new test cage and allowed to acclimate for at least 1 h. A novel animal was then introduced to the cage for 5 min during which the animals were allowed to freely interact. The social exposure occurred during the dark phase of the circadian cycle (6–9 pm). The interaction was video recorded under red light conditions with an infrared camera and score by hand using JWatcher^1^ for three types of behaviors: nape attacks, pinning and supine. Social play behaviors were categorized based on published descriptions of rat social play (Veenema and Neumann, 2009).

#### Social Recognition

Adult males of each genotype (WT and KO) were tested for social investigation and recognition of 12 juvenile male rats (5–7 weeks, weighing a minimum of 100 g less than the adults). Experimental subjects were placed in individual test cages and all animals were acclimated to the test room for 1 h prior to testing. The juvenile was then introduced to the test cage and allowed to freely interact with the experimental subject for 4 min. The juveniles were then removed and the animals were separated for 30 min before the juveniles were re-introduced for a second 4-min investigation period. Both investigations were video recorded and hand-scored for time spent in olfactory investigation of either the head or anogenital area of juveniles using JWatcher.

#### Social Approach

Adult male rats of each genotype (WT and KO) were tested for social approach in a three-chambered test arena. The custom built test chamber was 60 × 30 × 20 cm (W × D × H). The corrals were made from clear PVC pipe (5” diameter) with holes (1”) drilled throughout. Each animal was placed in the center arena for 5 min and allowed to explore the entire arena for an additional 10 min in the absence of conspecifics. A corral encaging an adult male conspecific was then introduced to one of the two outer chambers and an empty corral was introduced to the other chamber. The experimental animal was then allowed to explore the entire arena again for 10 min. Finally, a novel animal was added to the previously empty corral and the experimental animal had another 10-min exploration period. All stimulus animals were previously acclimated to the corrals to minimize stress-induced behaviors. Time the experimental animal spent investigating each chamber and the total distance traveled was scored using CleverSys TopScan (CleverSys Inc., Reston, VA, USA).

#### Open Field Activity and Repetitive Activity

Adult male rats of each genotype (WT and KO) were acclimated to the test room for 1 h and then place individually in a VersaMax AccuScan locomotion chamber for a 1-h test period. The animals were allowed to freely move around an unlit chamber, and their location was tracked by the disruption of IR beams. The total distance traveled, the time spent in the center area, vertical rearing and circling (both clockwise and counterclockwise) was interpolated through the pattern of beams broken based on the manufacturer programmed settings.

#### Operant Conditioning and Progressive Ratio Response

Eight male rats of each genotype (WT and KO) were food-restricted to 80%–85% of their free feeding body weight and introduced to a MedAssociates (Fairfax, VT, USA) dual lever, pellet reward operant response chamber. Animals were acclimated to the chamber and food reward (sucrose diet pellets) for 1 h on the first day of training. On the second day of training, the response levers were introduced such that one lever was paired with food reward delivery (“active” lever) and one was unpaired (“inactive” lever) at an FR1 schedule modified to elicit response at both levers. Training continued until 80 responses were obtained during a 60-min trial for each group. All animals were then advanced to a progressive ratio reward schedule during which the number of lever presses required to obtain a reward increased non-linearly with each subsequent trial. The number of presses on the active lever and the inactive lever was recorded. The breakpoint was defined as the number of lever presses per single reward at which the animals were unwilling to work. The breakpoint was used as a measure of motivation. The number of lever presses on the active vs. inactive lever was used as a measure of goal directed vs. hyperactive behavior (goal directed behavior should be specific to the active lever only). For one testing session the animals were allowed to consume the reward pellets to satiety for 1 h prior to testing as opposed to the typical conditions in which the animals only had access to a standard rat diet. The breakpoint was compared between the “reward sated” session and the typical “reward hungry” sessions as a measure of the sensitivity of breakpoint to reward value. For three additional testing sessions, each 1 week apart, the animals were treated with the dopamine D1 receptor antagonist SCH-39166 (0, 0.01, 0.1 mg/kg, I.P.) in a randomized fashion and the effect of the pharmacological manipulation on breakpoint was measured.

#### Location Discrimination Touchscreen Task

Eight WT and five KO male rats were food restricted to 80%–85% of their free feeding body weight. Equal groups were used initially but three of the KO failed to acquire the early testing behaviors and were removed from the study. Rats were then habituated to the touchscreen chamber and the behavior of the animal was shaped such that the animal learned to touch the illuminated stimulus on the screen. This pretraining phase is composed of five stages in which animals progressed through training in a criteria-dependent manner. After this period, the rats advanced to the acquisition of the location discrimination reversal task in which animals were required to make seven correct touches to a response window in eight consecutive trials. Once this criterion was met, the rule changed and the opposite location was then assigned as correct and was rewarded (total of 50 trials or 60 min time point). This acquisition phase was continued until an animal achieved the initial acquisition followed by a reversal in three of four consecutive sessions.

#### Pharmacological Modulation of Open Field Activity and Repetitive Activity

For behavioral pharmacological experiments, Cohort 4 was used for the dopamine D1 receptor antagonist (SCH-39166 0, 0.01, or 0.1 mg/kg, I.P.) open field experiment using a randomized within-subjects design (*N* = 10–11/genotype). Cohort 5 was used for the mGluR1 receptor antagonist (JNJ16259685 0 or 0.63 mg/kg, S.C.) open field and social behavior experiment using a randomized within-subjects design (*N* = 10/genotype). Animals received a subcutaneous injection 30 min prior to testing. Testing was conducted in the pharmacological experiments as described above.

### Synaptic Protein Analysis

#### Proteomic Analysis of Brain Homogenate

Adult animals were anesthetized via CO_2_ and rapidly decapitated (*N* = 4/genotype). The hippocampus (HP) and striatum were rapidly dissected out and frozen in liquid N_2_, and brain samples were kept at −80°C until assayed. Subcellular fractionation of the samples was then performed to isolate synpatosomal fractions and western blot interrogation of synaptic proteins expression was done as previously described with minor modifications (Schmeisser et al., 2012; Distler et al., 2014). Briefly, the sections were homogenized in HEPES-buffered sucrose and separated into synaptosomal fractions. Equal amounts of protein 10–20 μg were separated by SDS-polyacrylamide gel electrophoresis and then blotted onto polyvinylidene fluoride membranes. The membranes were incubated with specific primary antibodies and then visualized with HRP-conjugated secondary antibodies on X-ray film. The films were imaged and the gray value was quantified using ImageJ and normalized to the gray value of actin.

### Anatomical Analysis

Adult animals (*N* = 5/genotype) were anesthetized via CO_2_ and then rapidly decapitated. Brains were removed, fixed in 10% formalin, and divided into rostral/caudal blocks and stained by the rapid Golgi variant by Neurostructural Research^2^ as previously described (Valverde, 1993). Coronal sections (120 μm) were mounted, and striatal MSNs and HP CA1 pyramidal cells were imaged using a Zeiss bright field microscope. Camera lucida drawings were prepared from *n* = 4–7 randomly selected neurons per brain that met selection criteria (well-stained, arbor not obscured by other anatomy, soma in the middle third of the tissue section, and the appearance of spines). Neuronal morphology was quantified using Sholl and branch point analyses of the basilar arbor of hippocampal CA1 pyramidal (HPp) cells and the full arbor of MSN. The soma size of all neurons were also analyzed. Results were generated from values averaged across individual neurons per animal (WT: *N* = 5, *n* = 32 neurons; KO: *N* = 5, *n* = 31 neurons).

### Electrophysiological Characterizations

#### *In Vitro* Slice Preparation

Acute slices (300 μm) were prepared from adult male and female animals of each genotype (WT and KO). The results from both male and female animals were statistically compared, found to be not different and then pooled to increase statistical power. Rats were anesthetized with isoflurane (4%) and transcardially perfused prior to decapitation with oxygenated ice-cold artificial cerebral spinal fluid (aCSF) containing (in mM): NaCl, 125; NaHCO_3_, 25; glucose, 10; KCl, 3.5; NaH_2_PO_4_, 1.25; CaCl_2_, 0.5; MgCl_2_, 3; pH 7.4, osmolarity 295 mOsm, constantly oxygenated with 95% O_2_ and 5% CO_2_. Coronal slices containing the dorsal HP or sagittal slices containing the striatum and preserving corticostriatal fiber tracts were sectioned using a Vibratome. Slices were incubated in oxygenated aCSF warmed to ~34°C for at least 1 h prior to recording. Field excitatory postsynaptic potentials (fEPSPs) or whole-cell patch-clamp recordings were performed. For each experiment, individual slices were placed in a submersion-type recording chamber superfused with oxygenated aCSF (fEPSP flow rate of 2.5 mL/min; whole-cell flow rate of 2 mL/min) and maintained at 33–34°C. Recording aCSF formulations were adjusted to include 2 mM CaCl_2_ and 1 mM MgCl_2_.

#### *In Vitro* Field Recordings in Hippocampus

Field EPSPs were recorded using a multi-channel recording system (MED64, Alpha MED Scientific). Slices were transferred to one of four MED-P515A recording chambers containing 64 planar micro-electrodes arranged in an 8 × 8 grid embedded in the center of a transparent glass cylinder (10 mm depth). The recording chambers were connected to a 64-channel amplifier which was divided using a 4-way splitter such that four slices were recorded simultaneously. Each slice was in a separate chamber with 16 channels active in an 8 × 2 configuration. Each electrode across all chambers corresponded to a single amplifier channel. This orientation provided each electrode the ability to deliver stimuli as well as record electrophysiological signals. fEPSPs were evoked in the Schaffer collateral fiber pathway in the CA1 region with a biphasic square-wave pulse (0.20 ms; 0.05 Hz) delivered through one electrode. Only those electrodes positioned beneath the stratum radiatum of the CA1 region of the HP were utilized for stimulation or recording. Electrophysiological signals were high-pass filtered at 0.1 Hz and digitized at 20 kHz using MED64 Mobius acquisition software (WitWerx Inc., Santa Cruz, CA, USA).

To characterize the synaptic input-output relationship, fEPSPs were measured at fixed increments between 10–100 μA in slices from WT female *N* = 2 animals, WT male *N* = 6 animals, KO female *N* = 3 animals, KO male *N* = 6 animals. The stimulus intensity for the remainder of the session was set at a level that elicited ~50% of the maximum fEPSP response (20–50 μA). Changes in synaptic plasticity were examined following a 10 min stable baseline recording. Long-term potentiation (LTP) was induced with a high frequency theta burst stimulation (TBS; 5 trains of 4 pulses at 100 Hz repeated twice) given at the same stimulus intensity used for the 0.05 Hz test stimuli in slices from WT female *N* = 5 animals, WT male *N* = 5 animals, KO female *N* = 5 animals, KO male *N* = 4 animals. Long-term depression (LTD) was induced using a paired-pulse low frequency stimulation (PP-LFS) protocol, which included 900 paired stimuli (50 ms interstimulus interval) delivered at a 1-Hz frequency (Kemp and Bashir, 1999) in slices from WT male *N* = 6 animals, KO male *N* = 6 animals. Peak fEPSP amplitudes were calculated using the MED64 hardware and software packages. The percent change in amplitude was measured by comparing the mean fEPSP amplitude during the 10 min baseline with the mean fEPSP amplitude during the last 10 min of recording.

#### Whole-Cell Patch Clamp Recordings

Whole-cell voltage clamp recordings were performed from pyramidal cells within the CA1 region of the HP (WT female *N* = 11 cells/5 animals; WT male *N* = 9 cells/7 animals; KO female *N* = 10 cells/5 animals; KO male *N* = 8 cells/6 animals) or MSNs in the ventral striatum (WT male *N* = 11 cells/7 animals; KO male *N* = 10 cells/8 animals. Cells were identified using infrared differential interference contrast (IR-DIC) video microscopy (Olympus BX50-WI) using a 40× water-immersion objective. Visual guidance was obtained with an IR-sensitive CCD camera (DAGE-MTI) connected to a monitor. Patch pipettes (6–10 MΩ) were made from 1.5 mm O.D. boroscilicate glass tubing (World Precision Instruments, Sarasota, FL, USA) and filled with (in mM): CSCH_3_SO_3_, 126; HEPES, 10; MgCl_2_ 2; CsCL, 20; Spermine, 0.1; QX-314, 1; Mg-ATP, 2; Na_2_-ATP, 2; and GTP, 0.3; EGTA, 8; pH 7.3; osmolarity 280 mOsm. In a subset of cells Neurobiotin 488 (0.125%) was added to the internal recording solution for histological identification of recorded cells. Whole-cell recordings were acquired with a computer-controlled Multiclamp 700B amplifier (Axon Instruments, Foster City, CA, USA), digitized (Digidata, Axon Instruments) and sampled with Axoscope 9.0 (Axon Instruments) at a rate of 10 kHz. Electrode potentials were adjusted to zero before recording without correcting the liquid junction potential. Experiments began after reaching a steady resting state (approximately 5 min) and input resistance was monitored continuously with a 5 mV hyperpolarizing pulse (500 ms) given with every afferent stimulus.

Excitatory postsynaptic currents (EPSCs) were evoked using a bipolar electrode made from a twisted pair of Teflon-coated tungsten wires (tips approximately 200 μm apart; FHC, Bowdoin, ME, USA). In all whole cell experiments, afferent stimulation was elicited at a baseline frequency of 0.066 Hz. Neurons were voltage-clamped at a membrane potential of −70 mV except where noted. For hippocampal AMPA/NMDA ratio experiments, the stimulating electrode was placed in the Schaffer collateral pathway in the most lateral aspect of the CA1 region, no less than 500 μm from the recorded pyramidal cell in the presence of picrotoxin (10 μM). Upon establishing a steady baseline (10 min), the cell was held at −80 mV for 10 sweeps to record fast AMPA-mediated currents evoked by electrical stimulation. The cell was then held at +40 mV and fast AMPA- and slower NMDA-mediated synaptic currents were recorded for 10 min. Subsequently, the AMPA EPSC was isolated at +40 mV via bath application of the NMDA receptor antagonist DL-AP5 (100 μM). The AMPA to NMDA ratio was determined by dividing the peak of the response at −80 mV (AMPA) by the value obtained subtracting the response 40 ms following stimulation at +40 mV before and after DL-AP5 (NMDA; Counotte et al., 2014). For corticostriatal LTD experiments, the stimulating electrode was placed in the white matter at the PFC-striatum border. Following steady baseline, LTD was induced by pairing low frequency presynaptic stimulation (p-LFS; 1 Hz, 480 pulses) with postsynaptic depolarization by holding the membrane potential at −50 mV. Similar to extracellular recordings, the percent change in amplitude was measured by comparing the mean fEPSP amplitude during the 10 min baseline with the mean fEPSP amplitude during the last 10 min of recording. All fEPSP and EPSC amplitudes were averaged per minute with the exception of AMPA/NMDA values, which represent the average of 10 consecutive sweeps.

#### *In Vivo* Field Potential Recording

Rats of each genotype (*n* = 8 KO and *n* = 7 WT) were surgically implanted with titanium recording screws located over the left medial frontal cortex (AP +0.15 cm, ML +0.15 cm relative to Bregma) and the left parietal cortex (AP −0.37 cm, ML +0.22 cm relative to Bregma) for recording and screws over the left cerebellum and the right cerebellum for reference and grounding, respectively. The screws were implanted stereotaxically under isoflurane (2%) anesthesia and wired to a custom electronic interface board (EIB; ADPT-HS-18-Push Pin; Neuralynx Inc., Bozeman, MT, USA). The EIB connected via an Omnetics connector to the HS-18 headstage preamplifier and data was acquired using a Neuralynx Digital Lynx Data Acquisition System. After a minimum of 1 week of surgical recovery, the animals were acclimated to the recording chambers and the tethering procedure for three sessions. Recording experiments began at least 3 weeks after surgery to allow for recovery of neural tissue and receding of inflammatory response. During each recording session animals were allowed to habituate to the recording cage for 30 min and then signal quality was assessed for an additional 30 min. Baseline signal was recorded for 30 min and then auditory evoked responses were recorded in response to a white noise tone at 70 db, 80 db and 90 db. The tones were presented in a paired pulse paradigm in a train of 200 tones with 500 ms between the paired pulses and 10 s between each trial. The baseline signal was analyzed for spectral power using the fast Fourier transform with a 1.024 s Hanning window resulting in 0.9766 Hz resolution using Spike 2 (CED Cambridge, UK). Event related signals were extracted in a window from 100 ms pre-stimulus to 700 ms post-stimulus relative to the first tone to encompass the response to both tones. Average waveforms were calculated for each subject by averaging the 800 ms window across the 200 trials. Waveform features including P1, N1, P2 and N2 amplitude and latencies were calculated from the averaged waveform for each subject. P1 suppression was calculated as the ratio of P1 after tone 1 to P1 after tone 2 for each subject. Grand averaged waveforms for each genotype were generated by averaging the averaged waveforms for each subject.

## Results

The stastical comparisons, results, means, SEM and groups for each result described in the text are enumerated in Supplementary Table S1.

### Model Generation

Shank2 KO rat lines were generated at Horizon Discovery by targeting exon 31 for deletion by zinc finger nuclease technology. Two lines, 8A and 13 were confirmed by sequencing (data not shown). Line 13 has a 437 bp deletion around and including the entire exon 31, thereby causing a frameshift and premature stop codon in all three known isoforms of the rat Shank2 mRNA (Figure 1A). Deletion was confirmed at the genomic (Supplementary Figure S1A), mRNA (Figure 1B), and protein level (Figure 1C and Supplementary Figure S1B). Changes in the rat Shank2 mRNA was confirmed by RT-PCR using primers E30: 5′-GAGGGCTTTGGATTTGTG-3′ and E32: 5′-GACGACCTTAAGGACGAG-3′, with the expected PCR products for WT and KO mRNA of 248 bp and 123 bp, respectively (Figure 1B; arrows showing expected WT and KO PCR products). SHANK2 deletion was confirmed in synaptosomes and whole brain lysates via western blot using an antibody raised against the targeted SH3/PDZ domain of the protein (Figure 1C).

### Shank2 KOs Social Deficits Across Development

**Figure 2:**
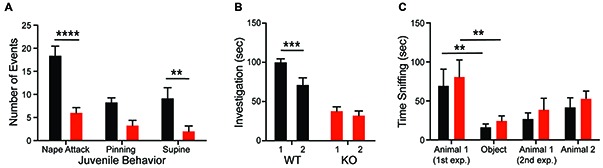
*Shank2* mutation results in impaired social behavior. *Shank2* KOs engage in less social interaction as juveniles **(A)** and as adults **(B)**. The decreased social interaction impairs social recognition **(B)** but not social preference **(C)**. In all panels WT animals are in black and KO animals are in red. Bars indicate SEM. **p < 0.01, ***p < 0.001, ****p < 0.0001.

Consistent with the hypothesis that motivational abnormalities in autism underlie social impairments (Chevallier et al., 2012), KO rats also demonstrate deficits in social interactions across development. Compared to WTs, KO animals fail to both initiate play behaviors (nape attack) and respond appropriately to social solicitation (supine) when interacting with a WT juvenile conspecific (Figure 2A; *genotype*: *F*_(1,14)_ = 28.32, *p* = 0.0001). The KO deficit is maintained into adulthood as evidenced by a decrease in olfactory investigation of a juvenile animal, commensurate with a lack of social recognition. As expected, WT animals showed a decrease in olfactory investigation time during re-exposure to a familiar conspecific after a 30-min delay; however, investigation by KO animals does not change (Figure 2B; *genotype*: *F*_(1,24)_ = 38.5, *p* < 0.0001). In the three-chambered social investigation paradigm, however, KO animals show a preference for a novel animal over a novel object as measured by time with nose in proximity to the stimulus cup, similar to WT animals (Figure 2C; *stimulus*: *F*_(3,54)_ = 10.2, *p* < 0.0001). While both groups also show a preference for the novel animal over a familiar animal, this effect is not significant for either genotype. There are also no significant differences in time spent in each chamber between the genotypes.

### Shank2 KOs Exhibit Hyperactivity and Repetitive Behavior

**Figure 3:**
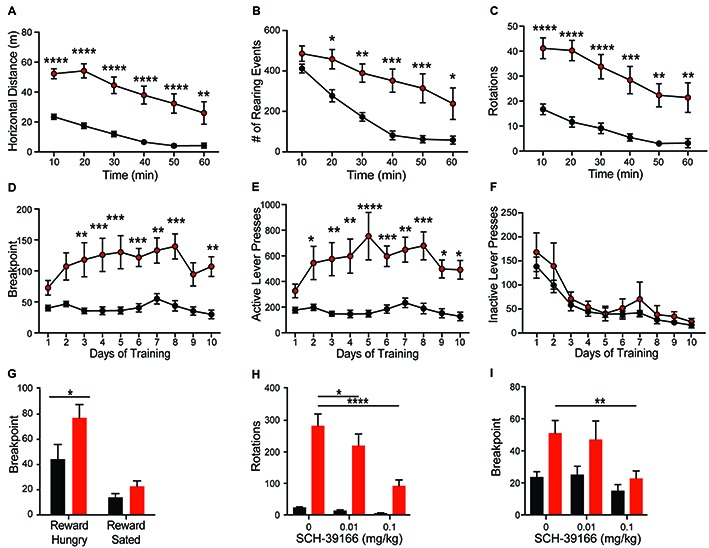
*Shank2* mutation results in increased locomotion, stereotypic and motivated behavior. *Shank2* KOs exhibit increased locomotion **(A)**, vertical rearing **(B)**, and rotations **(C)** in the open field arena relative to WT animals. In the progressive ratio operant task, KOs have an increased breakpoint (number of lever presses at which the animal will cease to work for a fixed reward) **(D)**. The increased response is specific to the active lever **(E)**, not the inactive lever **(F)**, suggesting the increased breakpoint is motivationally driven. Devaluation of the reward through pre-feeding to satiety prior to testing decreases the breakpoint of both WT and KO rats **(G)**. Both the repetitive circling **(H)** and increased breakpoint **(I)** can be reduced by administration of the D1 receptor antagonist SCH-39166. In all panels WT animals are in black and KO animals are in red. Bars indicate SEM. *p < 0.05, **p < 0.01, ***p < 0.001, ****p < 0.0001.

Consistent with the dominant phenotype seen in *Shank2* mouse models (Schmeisser et al., 2012; Won et al., 2012), we observe hyperactivity in Shank2 KO rats. Open field analysis reveals increased horizontal distance traveled (Figure 3A; *genotype F*_(1,20)_ = 32.46, *p* < 0.0001) and decreased thigmotaxis (graph not shown; *F*_(1,20)_ = 6.743, *p* < 0.0001) relative to WTs. Decreased thigmotaxis, though, cannot be separated from general increase in locomotion in these analyses. KOs also exhibit increased rearing (Figure 3B; *genotype F*_(1,20)_ = 15.81, *p* < 0.001) and repetitive circling (Figure 3C; *genotype* *F*_(1,20)_ = 25.57, *p* < 0.0001). These latter phenotypes are unique to the rat mutant model and are not seen in mouse models of Shank2 mutation. KOs engage in ~10-fold more rotations than WT animals, with no orientation bias (not shown). Importantly, KOs also show a similar rate of habituation to the chamber as WT animals, but never return to WT levels in any locomotor behavior.

### Deletion of Shank2 Results in a Hypermotivation Phenotype

Hyperactivity and repetitive behaviors are attributed to disruption of striatal motivational circuits (Kim et al., 2016; Szechtman et al., 2017). To explore whether repetitive phenotypes in *Shank2* mutant rats are associated with increased motivated behavior, animals were assessed in a progressive ratio operant response task. KO rats exhibit a significantly higher breakpoint than WTs (Figure 3D; *genotype F*_(1,14)_ = 17.83, *p* = 0.0009). The increase in response is specific to the reward-associated lever (Figures 3E,F; active, *genotype F*_(1,14)_ = 19.21, *p* = 0.0006; inactive, *F*_(1,14)_ = 0.756, *p* = 0.3992), and devaluation of the reward by pre-feeding to satiety decreases response in KO rats similarly to other genotypes (Figure 3G; reward hungry vs. reward sated; *genotype F*_(1,14)_ = 4.664, *p* = 0.0486). Together, these behaviors suggest an enhancement in motivated response independent of hyperactivity.

### D1 Dopamine Receptor Regulation of Both Hypermotivated and Repetitive Phenotypes

Enhancement of striatal D1 receptor (D1R) signaling, particularly within the ventral striatum, contributes to hyperactivity, repetitive behavior, and motivated behavior (Aberman et al., 1998; Shi and McGinty, 2011). To test the sensitivity of *Shank2* mutation-induced behaviors with dopamine receptor modulation, we evaluated the effect of the selective D1/D5R antagonist SCH-39166 on both repetitive circling and the hypermotivated response in progressive ratio. SCH-39166 dose-dependently reduces the production of repetitive circling in KO animals (Figure 3H; *dose*: *F*_(2,38)_ = 22.86, *p* < 0.0001; *dose × genotype*: *F*_(2,38)_ = 16, *p* < 0.0001). A Sidak’s *post hoc* test shows an effect of drug in the KO animals (vehicle vs. 0.01 mg/kg SCH-39166, *p* = 0.0168; vs. 0.1 mg/kg, *p* = 0.0001); however, in WT animals, *p* > 0.05 for both comparisons. Similarly, 0.1 mg/kg of SCH-39166 also decreases the breakpoint in progressive ratio (Figure 3I; *dose* *F*_(2,28)_ = 5.033, *p* = 0.0136). A Sidak’s *post hoc* test shows an effect of drug in the KO animals (vehicle vs. 0.1 mg/kg SCH-39166, *p* = 0.0388); however, in WT animals, *p* > 0.05 for both comparisons. The inhibitory effect of the D1/D5R antagonist on the production of these behaviors is consistent with striatal dysregulation (Higa et al., 2017).

### Shank2 KO Animals Exhibit Paradoxical Task Acquisition Deficits

**Figure 4:**
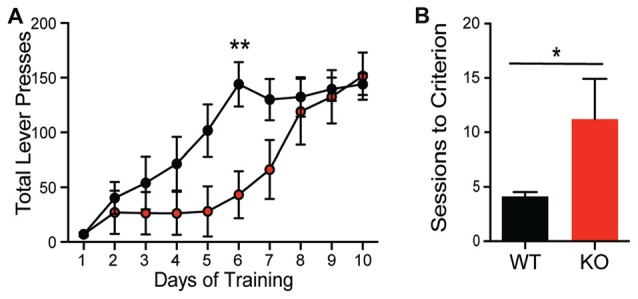
*Shank2* mutation results in impaired acquisition of operant behavior. *Shank2* KOs require more training to acquire both the operant lever press task **(A)** and a location discrimination touchscreen task **(B)**. In all panels WT animals are in black and KO animals are in red. Bars indicate SEM. *p < 0.05, **p < 0.01.

The differential role of Shank proteins in distinct brain regions may result in discordant behavioral phenotypes after *Shank2* mutation (Reim et al., 2017). Despite the hypermotivated response in progressive ratio, KOs take significantly longer to acquire the association between the lever press and reward during training using a fixed ratio (Figure 4A; *interaction F*_(9,126)_ = 2.832, *p* = 0.0046). Consistent with this learning deficit, KOs also exhibit a delay in acquisition in a location discrimination touchscreen task; significantly more trials are needed for the KO to learn the reward-action pairing (~11 days compared to 4 days for WT animals; Figure 4B; unpaired Student’s *t*-test; *p* = 0.033), with several KO animals never learning the association. The impairments in complex task acquisition suggest hippocampal memory alterations resultant of *Shank2* mutation.

### Opposing Anatomical Alterations in Striatum and Hippocampus

**Figure 5:**
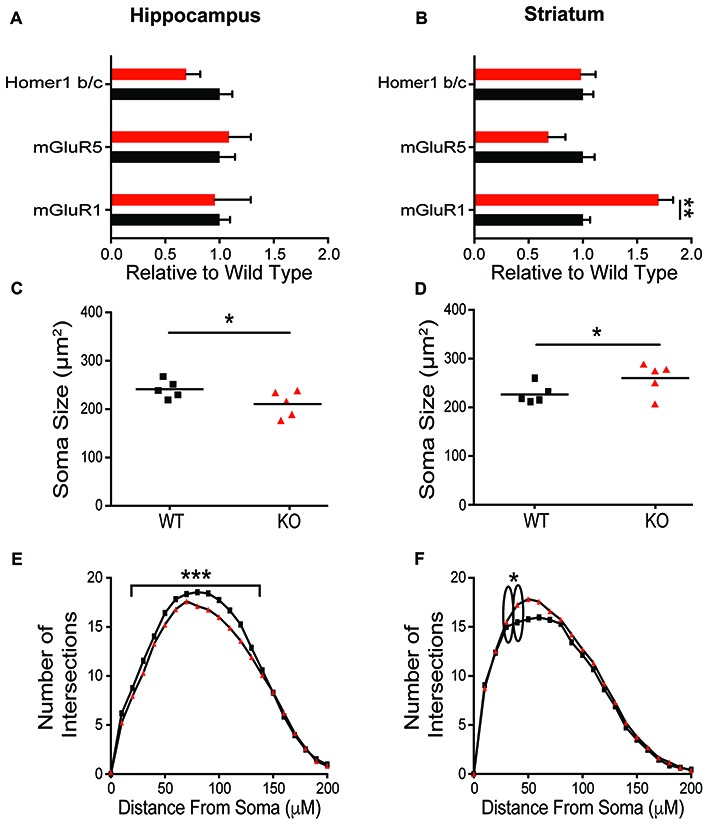
Differential molecular alterations in the hippocampus (HP) and striatum resultant of *Shank2* mutations. *Shank2* mutation does not alter the expression of synaptic proteins (Supplementary Figure S2) or metabotropic glutamate receptor related proteins in the HP **(A)**. Values are expressed as fold change relative to the average WT value. The soma size of CA1 hippocampal pyramidal (HPp) neurons is smaller in KO relative to WT rats **(B)** and there is a reduction in basilar dendritic arborization **(C)** as evidenced by Golgi staining and quantification. However, in medium spiny neurons (MSNs) of the striatum, detailed analysis reveals an upregulation of mGluR1 protein expression in male KO rats **(D)** despite a lack of change in synaptic protein level (Supplementary Figure S2). Striatal MSNs in contrast to HPps of KO rats have larger soma **(E)** and increased proximal dendritic branching **(F)** relative to WT animals. In all panels WT animals are in black and KO animals are in red. Bars indicate SEM. *p < 0.05, **p < 0.01, ***p < 0.001.

To explore the mechanisms contributing to the observed behavioral phenotypes, expression of postsynaptic proteins and cellular morphology in the HP and striatum were evaluated. In contrast to the dramatic behavioral phenotypic changes, there was no change in synaptic protein expression in the HP resulting from *Shank2* mutation in male rats (Figure 5A, Supplementary Figure S2). In HPp neurons, KO animals have smaller somas (Figure 5B; unpaired *t*-test, *p* = 0.0374) and decreased dendritic branching (Figure 5C; Wilcoxin rank-sum test, *p* = 0.0002) per animal (averaged across neurons). Consistent with the enhancement of behaviors mediated by the striatum and the impairment in operant learning, KO animals also exhibit reciprocally aberrant phenotypes in the anatomical alterations. KO rats have a significant upregulation of mGluR1 in the striatum compared to WT animals (Figure 5D; unpaired *t*-test, *p* = 0.0034), but no other significant changes in synaptic protein expression (Supplementary Figure S1). The MSNs of KO animals have larger neuronal somas (Figure 5E; unpaired *t*-test, *p* = 0.0477) and increased branching of the proximal dendritic arbor (Figure 5F; Wilcoxin rank-sum test, *p* < 0.0001).

### Functional Impairment in Hippocampal and Striatal *in Vitro* Physiology

**Figure 6:**
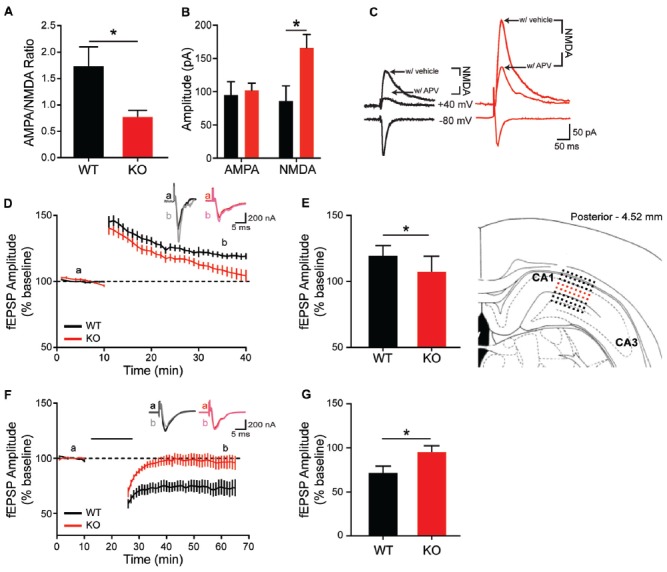
Altered *in vitro* hippocampal physiology of the *Shank2* KO rat. In the CA1 region of the HP, there is a reduction of the ratio of AMPA/NMDA synaptic currents **(A)** that is driven by an increase in the NMDA current **(B,C)**. KO rats also fail to maintain long-term potentiation (LTP) (**D,E**; brain slice depicts location of field recording), with significantly smaller fEPSPs 30 min after theta burst stimulation **(E)**. Inlaid traces are representative fEPSPs from the indicated time periods. KO rats also fail to achieve long-term depression (LTD) **(F)** with significantly larger field potentials 40 min after paired pulse low frequency stimulation (indicated by black bar) **(G)**. Bars indicate SEM. *p < 0.05.

To determine whether morphological and molecular alterations in hippocampal and striatal neurons are accompanied by changes in synapse physiology, extracellular and whole-cell patch clamp recordings were conducted in both brain regions. In the HPp, input-output curves do not differ between genotypes (data not shown; *F*_(9,135)_ = 0.2655, *p* = 0.98), indicating no change in basal synaptic transmission in the SC-CA1 pathway. However, there is a difference in the ratio of AMPA/NMDA currents within the HPp. Whole-cell voltage clamp recordings of HPp neurons indicate the alterations in hippocampal plasticity result from a reduction in AMPA/NMDA current ratio in KO rats (Figure 6A; unpaired *t*-test, *p* = 0.015), driven by an increase in NMDA-mediated current (Figures 6B,C; unpaired *t*-test, *p* = 0.0134). A failure is also observed in the maintenance of both synaptic potentiation and depression of evoked fEPSP amplitudes recorded in the HPp of KO animals. LTP induction results in a significant increase in synaptic response in WT (20 ± 2.22%), but not KO rats (8 ± 3.68%), observed 30 min post-stimulation (Figures 6D,E, unpaired *t*-test *p* = 0.0108). Similarly, LTD induction leads to an enduring reduction in fEPSP amplitude in WT (27 ± 6.67%), that is not observed in KOs (4 ± 6.00%) 40 min post-stimulation (Figures 6F,G; unpaired *t*-test, *p* < 0.025).

**Figure 7:**
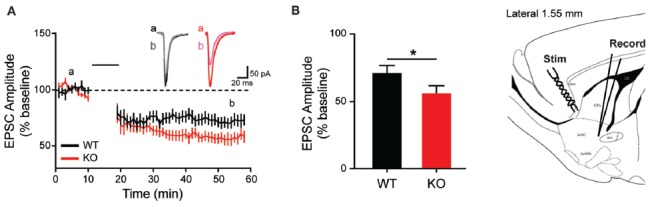
Altered long term depression in the striatum of the *Shank2* KO rat. In the striatum (brain slice depicts location of stimulating and recording electrode), KO rats show enhanced LTD **(A)** with increased depression relative to baseline 40 min after low frequency presynaptic stimulation (indicated by black bar) **(B)**. In all panels WT animals are in black and KO animals are in red. Bars indicate SEM. *p < 0.05.

Given the observed overexpression of striatal mGluR1 in male KO animals and the suggested role mGluR1 plays in LTD induction at excitatory synapses (Luscher and Huber, 2010), corticostriatal LTD is next examined. Consistent with behavioral and molecular findings, MSN recordings from KO animals show enhanced synaptic response (Figures 7A,B). Specifically, corticostriatal LTD induction results in significantly greater reduction of EPSC amplitude in KOs vs. WT (43 ± 4.75% vs. 28 ± 4.98%), measured at 40 min post-stimulation (Figure 7B; unpaired *t*-test, *p* = 0.0438). The enhanced LTD observed in mutant animals supports a pathological mechanism of increased activation of mGluR1 in the striatum.

### Characterization of *in Vivo* Electrophysiological Biomarker of Shank2 Mutation

**Figure 8:**
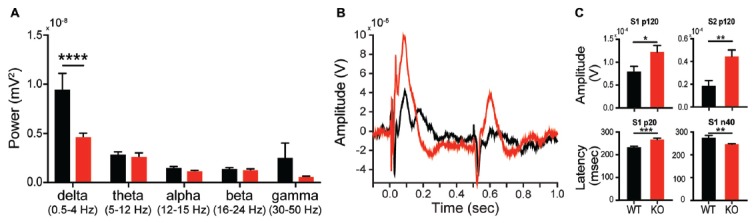
Altered *in vivo* electrophysiology of the *Shank2* KO rat. Alterations in neural responsiveness can be seen *in vivo*. KOs exhibit a reduction in spectral power in the delta frequency compared to WT animals during resting state conditions **(A)**. KOs have an altered evoked response to paired auditory stimuli **(B)**, with an increase in p120 amplitude and an increase in the latency of the p20 and n40 components of the cortical neural response **(C)**. In all panels WT animals are in black and KO animals are in red. Bars indicate SEM. *p < 0.05, **p < 0.01, ***p < 0.001, ****p < 0.0001.

Given the changes in synaptic plasticity we observed subcortically, we hypothesized a change in cortical processing accessible via *in vivo* neurophysiology would be present, thereby lending to a potential translational biomarker. EEG can be similarly collected in both rodent models and subjects with autism, making EEG-based biomarkers particularly amenable for preclinical to clinical translation (Jeste et al., 2015). During baseline conditions, analysis of EEG spectrum reveals a significant effect of genotype on spectral power (*interaction F*_(4,52)_ = 5.335, *p* = 0.0011; *genotype F*_(1,13)_ = 5.6692, *p* = 0.0329). This effect was driven by a significant reduction in parietal cortex delta (1–4 Hz) power in KOs compared to WTs (Figure 8A; Sidak’s test, *p* < 0.0001), however it is unknown if this is a state or a trait based phenomenon. Auditory evoked potentials are also altered in KOs relative to WTs (Figure 8B). At 80 db, the p120 amplitude for both S1 and S2 presentations is greater in KOs vs. WTs (Figure 8C; *p* = 0.044 and *p* = 0.0045, respectively). In KO animals, the early component latencies (p20 and n40) are significantly delayed for S1 and S2 (Figure 8C; S1-p20 *p* = 0.0008; S2-p20 *p* = 0.0481; Figure 8C, S1-N40 *p* = 0.0083; S2-N40 *p* = 0.0205). Similar effects are seen at both 70 db and 90 db (data not shown).

### Rescue of Striatal LTD and Repetitive Behavior by mGluR1 Antagonism

**Figure 9:**
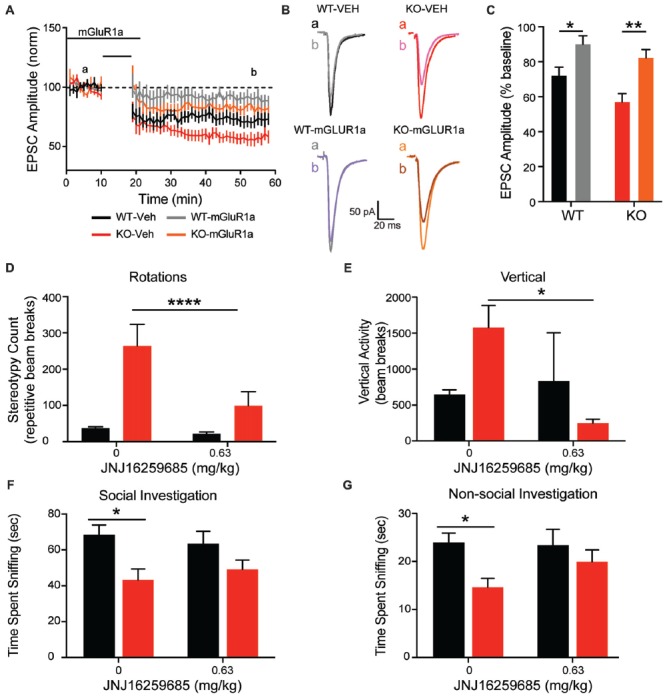
mGluR1 antagonist JNJ16259685 rescues both striatal physiology and behavior. Administration of the selective mGluR1 antagonist JNJ16259685 normalizes the enhanced striatal LTD response of the KO animals **(A)**. Traces are representative EPSPs from the indicated time periods **(B)**. Treatment significantly reduced the depression in both WT and KO rats **(C)**. Blocking mGluR1 receptors *in vivo* selectively rescues the striatally mediated stereotypic behaviors, including rotations **(D)** and rearing **(E)**, but does not have an effect on social **(G)** or non-social **(F)** investigation. In all panels WT animals are in black and KO animals are in red. Note the data in **(A)** for vehicle WT and KO data (black and red lines, respectively) are also depicted in Figure 7A. Bars indicate SEM. *p < 0.05, **p < 0.01, ****p < 0.0001.

To explore the association between increased mGluR1 receptor expression and enhanced corticostriatal LTD with aberrant repetitive behavior, the mGluR1 selective antagonist JNJ16259685 was administered *in vitro* and *in vivo*. In comparison to vehicle recordings depicted in Figures 7A, 9A (black and red lines for WT and KO, respectively), JNJ16259685 (100 nM) reduces corticostriatal LTD in both genotypes (Figures 9A–C; *treatment F*_(1,40)_ = 19.65, *p* < 0.0001 gray and orange lines for WT and KO, respectively). EPSC amplitude is increased by mGluR1 antagonist treatment (Figure 9C) in WT (*p* = 0.0169) and KO (*p* = 0.0015). After treatment, there is no significant difference between EPSC amplitude in vehicle treated WT rats and JNJ16259685 treated KO rats (*p* = 0.1551). Similar to D1-selective antagonism, administration of 0.63 mg/kg JNJ16259685 (~100% receptor occupancy (Lavreysen et al., 2004)) significantly reduces repetitive behaviors in the open field. In KO rats, both vertical rearing (Figure 9E; *interaction F*_(1,18)_ = 4.615, *p* = 0.456) and repetitive circling (Figure 9D; *interaction F*_(1,18)_ = 23.21, *p* = 0.0001; *dose F*_(1,18)_ = 33.8, *p* < 0.0001; Sidak *post hoc* *p* < 0.0001) are significantly decreased by mGluR1 antagonism, while no effect is seen in WT animals (Sidak *post hoc* *p* = 0.7042. Interestingly, the behavioral effect of JNJ16259685 is specific to repetitive behaviors, as there is no significant effect of drug on overall locomotion, social investigation, or novel object investigation (Figures 9F,G).

## Discussion

Consistent with analogous mouse models, *Shank2* mutation in rats results in hyperactivity and repetitive behaviors associated with striatal alterations. In addition to locomotor behaviors, our *Shank2* KO rats exhibit a novel hypermotivated response that is likely explained by striatal overexpression of mGluR1, altered neuronal morphology, and changes in MSN activity-dependent plasticity. Consistent with these findings, the mGluR1 antagonist JNJ16259685 normalizes striatal physiology and striatally mediated behaviors. In contrast to enhanced striatal function, *Shank2* mutant rats also demonstrate impairments in learning and decreased hippocampal plasticity and neuronal growth.

### Striatal Dysregulation in the Shank2 Mutant Rat

Hyperactivity and repetitive behaviors have been consistently observed across Shank-associated mouse models (Peca et al., 2011; Schmeisser et al., 2012; Won et al., 2012; Bariselli et al., 2016; Wang et al., 2016). The replicability of these phenotypes across mouse strains, and now species, makes repetitive behavior the most robust behavioral phenotype in Shank models (Jiang and Ehlers, 2013). Interestingly, our model also exhibits a hypermotivation phenotype that is also likely mediated by striatal function. Hyperactivity, repetitive behavior, and motivation have distinct underlying circuitries, however, all three are associated with striatal function, indicating a common node of pathophysiology (Britt et al., 2012). *Shank3* mutation disrupts the development of normal corticostriatal connectivity and ventral striatal maturation, implicating this family of proteins in the development of striatal circuits (Bariselli et al., 2016; Peixoto et al., 2016). While *Shank3* has been characterized as the “striatal” Shank protein, *Shank2* is also expressed in the rat striatum and may reciprocally regulate the expression of other SHANK proteins in this region (Böckers et al., 2004; Schmeisser et al., 2012; Han et al., 2013). In our model, striatal cellular morphology, physiology, and plasticity are disrupted following *Shank2* mutation. Remediation of striatal mGluR1 upregulation selectively rescues the repetitive but not the social phenotype, indicating the potential contribution of alternate mechanisms to different aspects autism symptomatology.

### Opposing Effect of Shank2 Mutation in Striatum and Hippocampus

In contrast to the enhancement of striatal activity, hippocampal function is depressed in Shank2 KO rats structurally, with decreased dendritic branching and soma size; cellularly, demonstrated by reduced synaptic plasticity; and behaviorally, as evidenced by impaired operant conditioning. Recent investigations into SHANK3 suggest regionally distinct functionality of SHANK proteins. Proteomic analysis of the PSD demonstrated *Shank3* mutation primarily disrupted glutamate receptor subunits in the striatum but cytoskeletal components in the HP (Reim et al., 2017). Interactome construction has also revealed a regional specificity of SHANK3 functionality that is not seen in its binding partners (Lee et al., 2017). Given the overlapping functionality within Shank proteins, Shank2 disruption may also result in comparable alterations. Nonetheless, further characterization is needed to determine whether region-specific functionality in Shank2 contributes to the opposing phenotypes described here.

### Mechanisms of Shank-Mediated Dysregulation

We show that *Shank2* mutation leads to several structural and functional changes in the striatum, including enhanced mGluR1 protein expression. Similar to our model, positive modulation of mGluR1a activity increases MSN dendritic spine density in ventral striatum (Gross et al., 2016). Consistent with these structural findings, we observe an enhancement of corticostriatal synaptic depression in *Shank2* mutant MSNs, in which mGluR1 is highly expressed in dendrites and spines (Testa et al., 1998). Activation of these postsynaptic receptors increases intracellular calcium (Pisani et al., 2000) through either polyphosphoinositide hydrolysis coupling (Casabona et al., 1997) or via activation of phospholipase C (Fagni et al., 2000). Given that increased postsynaptic calcium concentration is a critical mediator of striatal synaptic plasticity (Calabresi et al., 1992), this is a mechanism by which increased mGluR1 expression may enhance corticostriatal LTD. These data, together with loss of corticostriatal LTD following application of an mGluR1 antagonist (Figures 5A–C), support the assertion that striatal mGluR1 overexpression mediates aberrant striatal function and behavior in *Shank2* KO animals.

### Alteration of Glutamate Receptor Proteins After Shank2 Mutation

While the *Shank2* rat model shows only subtle, region-specific upregulation of mGluR1 protein, mouse models of *Shank2* mutation exhibit broad disruption of glutamate receptor subunit expression. *Shank2* transgenic mice have a dramatic upregulation of ionotropic receptors in the striatum and, to a lesser extent, the HP (Schmeisser et al., 2012). The differences in glutamate receptor dysregulation between these models are unclear, despite the use of identical protocols. However, the functional alterations in our rat model overlap with specific features of both *Shank2* mutant mouse models (Schmeisser et al., 2012; Won et al., 2012) but do not provide a consensus phenotype. The discordance of molecular but not behavioral phenotypes resultant from *Shank2* mutation in mice and rats suggests that alterations in glutamate receptor expression may not be a pathogenic locus of this mutation. Instead, changes in receptor expression profiles may be pathological manifestations that vary based on species, mutation, and environment resultant of an unidentified common mechanism.

### Importance of Circuit-Specific Modulation

Pharmacotherapeutic development for autism has primarily focused on treatments for social impairments, yet repetitive behaviors are a central component of ASD currently lacking FDA-approved treatments. Repetitive behaviors have greater translational face validity, are easier to quantify, and can be measured more objectively than social impairment in rodent models (Kaiser and Feng, 2015). Rescue of repetitive but not social phenotypes with mGluR1 inhibition in this study suggests the striatal circuit should be selectively modulated and may represent a tractable target for these symptoms. However, given the expression profile, mGluR1 inhibition to target striatal dysfunction could potentially lead to “off-circuit” effects. As the molecular alterations in our model are regionally specific, identifying circuit-selective modulators will be important. Regionally specific modulation of metabotropic glutamate receptors by SHANK protein mutation could be a source of the variability seen in the behavioral efficacy of drugs targeting these receptors. The opposing hippocampal and striatal alterations in our model suggest that circuit-selective methods of pharmacological manipulation are likely necessary for the successful treatment of the impairments in autism. This model uniquely enables the screening of novel compounds for both the desired repetitive behavioral effects and counter screening for cognitive effects.

### Benefits of Rat Transgenic Models

The characterization of human *Shank2* mutations in both rat and mouse models enables the assessment of species-specific preclinical model strengths. *Shank2* mutation in a rat model empowers more sophisticated characterization of the neural circuits and provides a better systems-level understanding of the relationship between synaptic dysfunction and behavioral phenotypes. Rats are also amenable to more complex surgical and electrophysiological manipulations due to their larger size. Further, there is greater homology in the metabolic pathways of rats and humans relative to mice and humans, making them the preferred animal model for drug development (Jaramillo and Zador, 2014; Ellenbroek and Youn, 2016; Homberg et al., 2017). Finally, more sophisticated behavioral endpoints can be assessed in rats due to their enhanced cognitive capabilities and diverse social behavioral repertoire, which is particularly relevant to the modeling of ASD-related mutations. Through the use of a rat genetic model, we have identified a unique hypermotivation phenotype and demonstrated social impairments early in development, extending the behavioral phenotypes previously seen in mouse models. These phenotypes have given credence to the hypothesis of autism as a disorder of motivation and striatal function (Chevallier et al., 2012) and inspire further investigation into the ability to selectively pharmacologically manipulate corticostriatal circuits for the treatment of ASD.

## Author Contributions

MM, JB, MS, TB, PO and DB conceptualized the experiments and edited the manuscript. MM, JB, EG, MB, RG and DR designed and executed the experiments and analyzed the data. MM, JB and DB wrote the manuscript.

## Conflict of Interest Statement

DB, JB, PO, MB, EG, RG and MM are former employees of Pfizer, Inc. MB and EG are currently employees of Biogen, Inc. PD and DB are currently employees of Takeda Pharmaceuticals. MM is currently at Harvard Medical School/Boston Children’s Hospital. JB is currently an employee at Alkermes, and RG is an employee of Axial Therapeutics. All were employed solely by Pfizer during the execution of the described experiments. The remaining authors declare that the research was conducted in the absence of any commercial or financial relationships that could be construed as a potential conflict of interest.

## Footnotes

^1^http://www.jwatcher.ucla.edu

^2^http://www.neurostructural.org

## Supplementary Material

FIGURE S1 Generation and validation of the Shank2 KO rat model. Genomic PCR strategy **(A)** determined gDNA sequence deleted in two rat Shank2 KO lines (8A and 13) including PCR strategy design and primers to detect deletions. DNA gel is example of WT, Het and KO animals’ genomic PCR results showing expected gPCR products of 1171 bp (WT) and 501 bp (KO). **(B)** Entire Shank2 western blot of whole brain and synaptosome preparations for WT, Het and KO animals from Figure 1C.

FIGURE S2 Lack of change in synaptic protein expression in *Shank2* KO rat. Neither the HP **(A)** nor the striatum **(B)** show significant changes in synaptic protein expression unlike in the *Shank2* mutnat mouse. Values are expressed as fold change relative to the average WT value. Bars indicate SEM.

TABLE S1 Comprehensive statistics table.
