# Supplementary material for: Hyperactivity and Hypermotivation Associated With Increased Striatal mGluR1 Signaling in a Shank2 Rat Model of Autism
Source: Front Mol Neurosci. 2018 Jun 19;11:107. doi: 10.3389/fnmol.2018.00107 (PMC6018399; doi:10.3389/fnmol.2018.00107)
Supplement: FIGURE S1 — Generation and validation of the Shank2 KO rat model. Genomic PCR strategy (A) determined gDNA sequence deleted in two rat Shank2 KO lines (8A and 13) including PCR strategy design and primers to detect deletions. DNA gel is example of WT, Het and KO animals’ genomic PCR results showing expected gPCR products of 1171 bp (WT) and 501 bp (KO). (B) Entire Shank2 western blot of whole brain and synaptosome preparations for WT, Het and KO animals from Figure 1C. [file Image_1.PDF]

a.

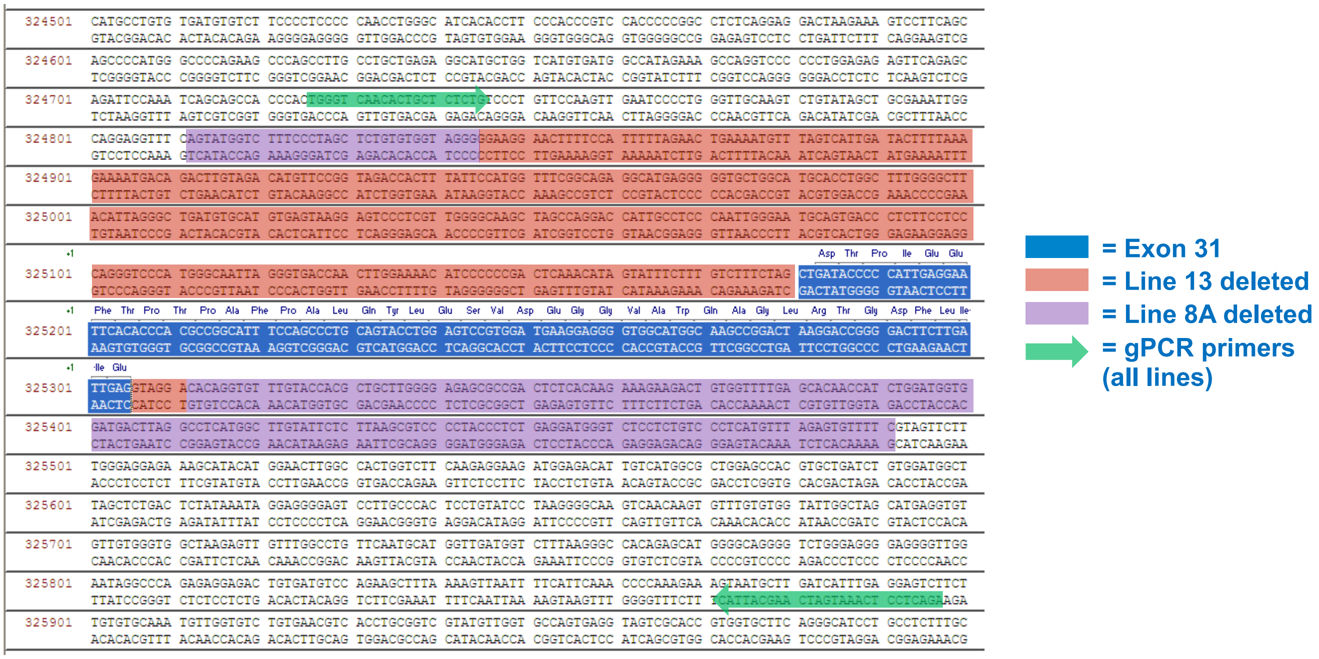
324501 CATGCCTGTG TGATGTGTCT TCCCCTCCCC CAACCTGGGC ATCACACCTT CCCACCCGTC CACCCCCGGC CTCTCAGGAG GACTAAGAAA GTCCTTCAGC
GTACGGACAC ACTACACAGA AGGGGAGGGG GTTGGACCCG TAGTGTGGAA GGGTGGGCAG GTGGGGGCCG GAGAGTCCTC CTGATTCTTT CAGGAAGTCG
324601 AGCCCCATGG GCCCCAGAAG CCCAGCCTTG CCTGCTGAGA GGCATGCTGG TCATGTGATG GCCATAGAAA GCCAGGTCCC CCCTGGAGAG AGTTCAGAGC
TCGGGGTACC CGGGGTCTTC GGGTCGGAAC GGACGACTCT CCGTACGACC AGTACACTAC CGGTATCTTT CGGTCCAGGG GGGACCTCTC TCAAGTCTCG
324701 AGATTCCAAA TCAGCAGCCA CCCACTGGGT CAACACTGCT CTCTGTCCCT GTTCCAAGTT GAATCCCCTG GGTTGCAAGT CTGTATAGCT GCGAAATTGG
TCTAAGGTTT AGTCGTCGGT GGGTGACCCA GTTGTGACGA GAGACAGGGA CAAGGTTCAA CTTAGGGGAC CCAACGTTCA GACATATCGA CGCTTTAACC
324801 CAGGAGGTTT CAGTATGGTC TTTCCCTAGC TCTGTGTGGT AGGGGGAAGG AACTTTTCCA TTTTTAGAAC TGAAAATGTT TAGTCATTGA TACTTTTAAA
GTCCTCCAAA GTCATACCAG AAAGGGATCG AGACACACCA TCCCCCTTCC TTGAAAAGGT AAAAATCTTG ACTTTTACAA ATCAGTAACT ATGAAAATTT
324901 GAAAATGACA GACTTGTAGA CATGTTCCGG TAGACCACTT TATTCCATGG TTTCGGCAGA GGCATGAGGG GGTGCTGGCA TGCACCTGGC TTTGGGGCTT
CTTTTACTGT CTGAACATCT GTACAAGGCC ATCTGGTGAA ATAAGGTACC AAAGCCGTCT CCGTACTCCC CCACGACCGT ACGTGGACCG AAACCCCGAA
325001 ACATTAGGGC TGATGTGCAT GTGAGTAAGG AGTCCCTCGT TGGGGCAAGC TAGCCAGGAC CATTGCCTCC CAATTGGGAA TGCAGTGACC CTCTTCCTCC
TGTAATCCCG ACTACACGTA CACTCATTCC TCAGGGAGCA ACCCCGTTCG ATCGGTCCTG GTAACGGAGG GTTAACCCTT ACGTCACTGG GAGAAGGAGG
+1 Asp Thr Pro Ile Glu Glu
325101 CAGGGTCCCA TGGGCAATTA GGGTGACCAA CTTGGAAAAC ATCCCCCCGA CTCAAACATA GTATTTCTTT GTCTTTCTAG CTGATACCCC CATTGAGGAA
GTCCCAGGGT ACCCGTTAAT CCCACTGGTT GAACCTTTTG TAGGGGGGCT GAGTTTGTAT CATAAAGAAA CAGAAAGATC GACTATGGGG GTAACTCCTT
+1 Phe Thr Pro Thr Pro Ala Phe Pro Ala Leu Gln Tyr Leu Glu Ser Val Asp Glu Gly Gly Val Ala Trp Gln Ala Gly Leu Arg Thr Gly Asp Phe Leu Ile
325201 TTCACACCCA CGCCGGCATT TCCAGCCCTG CAGTACCTGG AGTCCGTGGA TGAAGGAGGG GTGGCATGGC AAGCCGGACT AAGGACCGGG GACTTCTTGA
AAGTGTGGGT GCGGCCGTAA AGGTCGGGAC GTCATGGACC TCAGGCACCT ACTTCCTCCC CACCGTACCG TTCGGCCTGA TTCCTGGCCC CTGAAGAACT
+1 Ile Glu
325301 TTGAGGTAGG ACACAGGTGT TTGTACCACG CTGCTTGGGG AGAGCGCCGA CTCTCACAAG AAAGAAGACT GTGGTTTTGA GCACAACCAT CTGGATGGTG
AACTCCATCC TGTGTCCACA AACATGGTGC GACGAACCCC TCTCGCGGCT GAGAGTGTTC TTTCTTCTGA CACCAAAACT CGTGTTGGTA GACCTACCAC
325401 GATGACTTAG GCCTCATGGC TTGTATTCTC TTAAGCGTCC CCTACCCTCT GAGGATGGGT CTCCTCTGTC CCTCATGTTT AGAGTGTTTT CGTAGTTCTT
CTACTGAATC CGGAGTACCG AACATAAGAG AATTCGCAGG GGATGGGAGA CTCCTACCCA GAGGAGACAG GGAGTACAAA TCTCACAAAA GCATCAAGAA
325501 TGGGAGGAGA AAGCATACAT GGAACTTGGC CACTGGTCTT CAAGAGGAAG ATGGAGACAT TGTCATGGCG CTGGAGCCAC GTGCTGATCT GTGGATGGCT
ACCCTCCTCT TTCGTATGTA CCTTGAACCG GTGACCAGAA GTTCTCCTTC TACCTCTGTA ACAGTACCGC GACCTCGGTG CACGACTAGA CACCTACCGA
325601 TAGCTCTGAC TCTATAAATA GGAGGGGAGT CCTTGCCCAC TCCTGTATCC TAAGGGGCAA GTCAACAAGT GTTTGTGTGG TATTGGCTAG CATGAGGTGT
ATCGAGACTG AGATATTTAT CCTCCCCTCA GGAACGGGTG AGGACATAGG ATTCCCCGTT CAGTTGTTCA CAAACACACC ATAACCGATC GTACTCCACA
325701 GTTGTGGGTG GCTAAGAGTT GTTTGGCCTG TTCAATGCAT GGTTGATGGT CTTTAAGGGC CACAGAGCAT GGGGCAGGGG TCTGGGAGGG GAGGGGTTGG
CAACACCCAC CGATTCTCAA CAAACCGGAC AAGTTACGTA CCAACTACCA GAAATTCCCG GTGTCTCGTA CCCCGTCCCC AGACCCTCCC CTCCCCAACC
325801 AATAGGCCCA GAGAGGAGAC TGTGATGTCC AGAAGCTTTA AAAGTTAATT TTCATTCAAA CCCCAAAGAA AGTAATGCTT GATCATTTGA GGAGTCTTCT
TTATCCGGGT CTCTCCTCTG ACACTACAGG TCTTCGAAAT TTTCAATTAA AAGTAAGTTT GGGGTTTCTT TCATTACGAA CTAGTAAACT CCTCAGAAGA
325901 TGTGTGCAAA TGTTGGTGTC TGTGAACGTC ACCTGCGGTC GTATGTTGGT GCCAGTGAGG TAGTCGCACC GTGGTGCTTC AGGGCATCCT GCCTCTTTGC
ACACACGTTT ACAACCACAG ACACTTGCAG TGGACGCCAG CATACAACCA CGGTCACTCC ATCAGCGTGG CACCACGAAG TCCCGTAGGA CGGAGAAACG
= Exon 31
= Line 13 deleted
= Line 8A deleted
= gPCR primers (all lines)

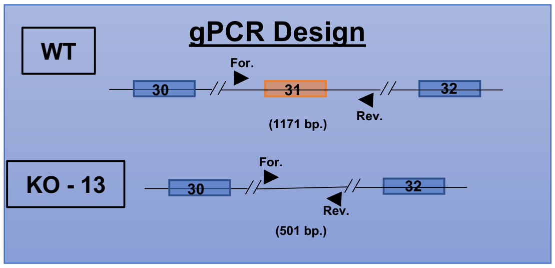
gPCR Design
WT
For.
30
31
32
Rev.
(1171 bp.)
KO - 13
For.
30
32
Rev.
(501 bp.)

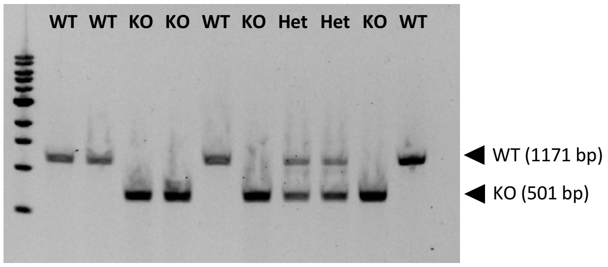
WT WT KO KO WT KO Het Het KO WT
WT (1171 bp)
KO (501 bp)

b.

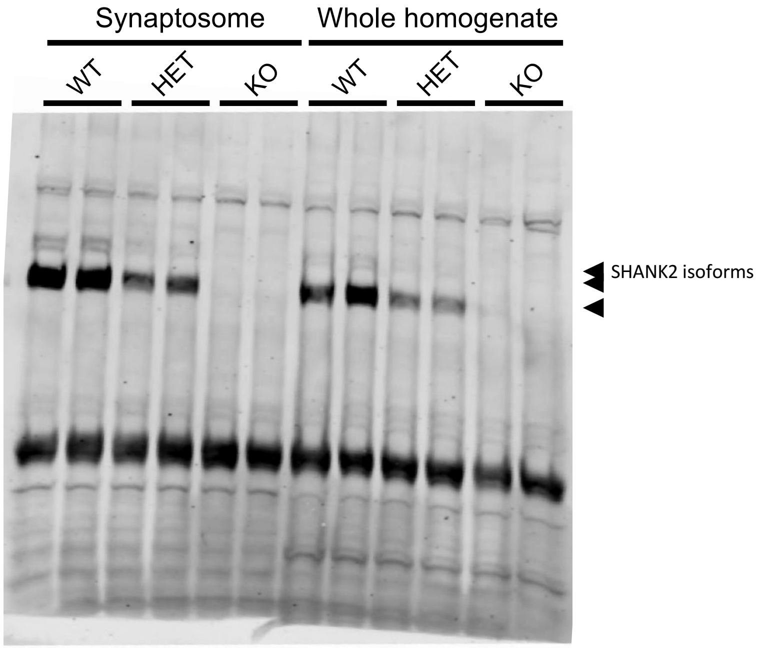
Synaptosome
Whole homogenate
WT
HET
KO
WT
HET
KO
SHANK2 isoforms
