# Supplementary figures and images for: Hyperactivity and Hypermotivation Associated With Increased Striatal mGluR1 Signaling in a Shank2 Rat Model of Autism
Source: Front Mol Neurosci. 2018 Jun 19;11:107. doi: 10.3389/fnmol.2018.00107 (PMC6018399; doi:10.3389/fnmol.2018.00107)

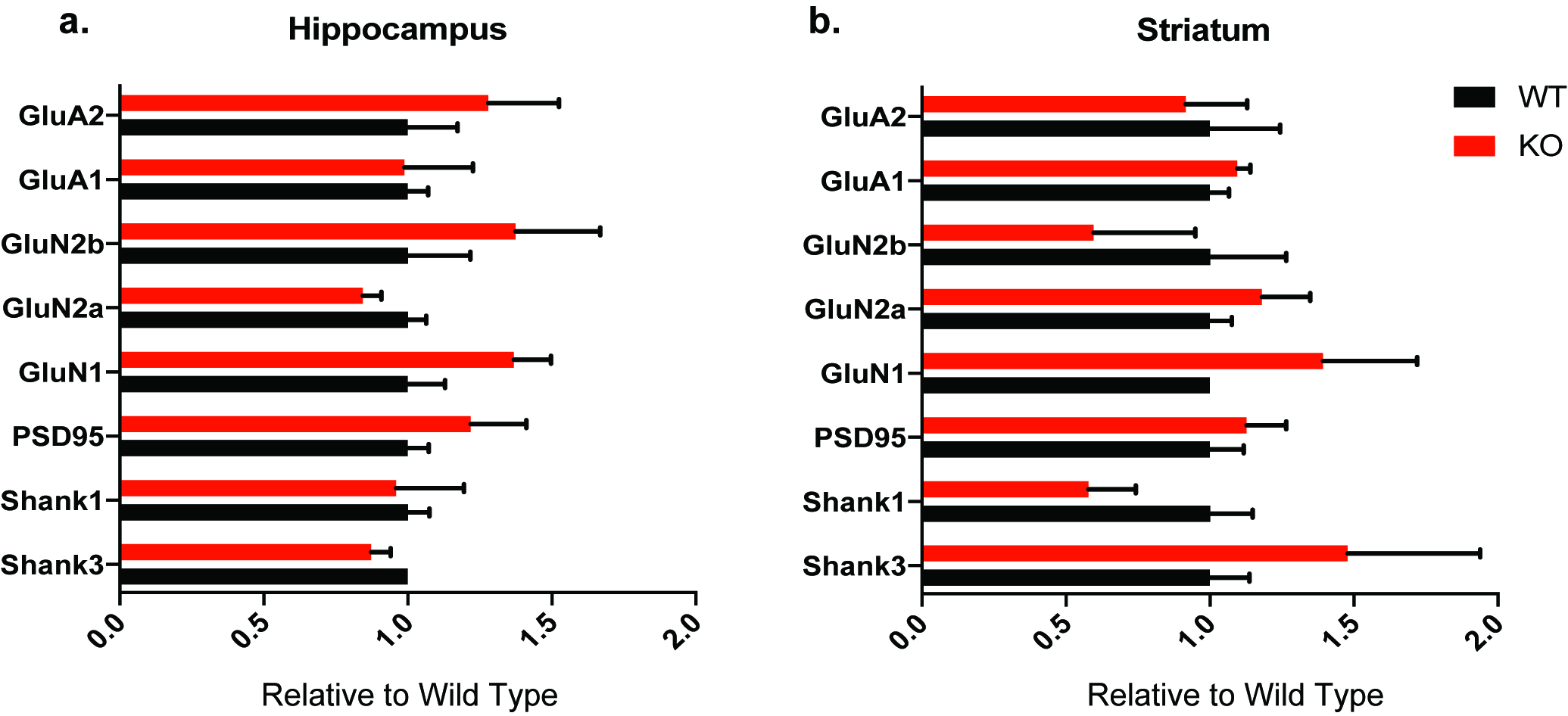

Supplement: FIGURE S2 — Lack of change in synaptic protein expression in Shank2 KO rat. Neither the HP (A) nor the striatum (B) show significant changes in synaptic protein expression unlike in the Shank2 mutnat mouse. Values are expressed as fold change relative to the average WT value. Bars indicate SEM. [file Image_2.TIF]
